# Supplementary material for: A randomized sham-controlled trial of transcranial and intranasal photobiomodulation in Japanese patients with mild cognitive impairment and mild dementia due to Alzheimer’s disease: a protocol
Source: Front Neurol. 2024 Jul 5;15:1371284. doi: 10.3389/fneur.2024.1371284 (PMC11258032; doi:10.3389/fneur.2024.1371284)
Supplement: Supplementary file 1 [file Data_Sheet_1.docx]

Information document on a randomized, double-blind, parallel-group comparative study of photobiomodulation for patients with dementia or mild cognitive impairment

Department of Psychiatry, National Center of Neurology and Psychiatry

Takuma Inagawa

table of contents

1. Introduction
2. The purpose and significance of this study
3. About this study
4. How this study will be conducted and the period of participation
5. Potential burdens, risks, and benefits of participating in the study
6. Other Treatment Methods
7. Financial burden associated with participating in this study
8. Regarding withdrawal of consent after participating in the study
9. Regarding the handling of personal information, etc.
10. Regarding storage and disposal of samples and information, and secondary use
11. Regarding the sources of research funding and conflicts of interest of researchers
12. Regarding provision of medical care after the research is conducted
13. Regarding handling of research results of those who participated in the study
14. Compensation for health damage caused by research participation
15. Disclosure of research information
16. If you would like to obtain or view materials related to the research plan and research methods
17. About the Institutional Review Board that reviewed this study
18. The implementation structure of this study, the names of the medical institutions conducting the study, and the name of the principal investigator
19. For inquiries regarding this research, please contact:

# 1. Introduction

This explanatory document has been prepared to help those considering participating in this clinical research to gain a deeper understanding when the principal investigator or co-investigator explains the details of the study to them.

After receiving an explanation of the research content and understanding the contents of this document, please decide whether or not to participate in the research. We ask that you decide of your own free will whether or not to participate in the research. As a result, we guarantee that you will not suffer any disadvantages as a result of not participating in the research. In addition, please understand that while the results of this research may generate intellectual property rights such as patents in the future, such rights will not belong to you as a research participant.

If you have any questions about the research, concerns, or anything else you would like to ask, please feel free to ask.

# 2. Purpose and significance of this study

Alzheimer's is a type of brain disease that affects the way people think, remember, and behave. It is very common in older adults and is the most common cause of dementia. Alzheimer's is caused by the buildup of amyloid plaques, a sticky protein found in the brain. These plaques are made of a protein called amyloid beta, which is normally flushed from the brain but can build up in people with Alzheimer's.

Over time, amyloid plaques damage brain cells and eventually cause their death. This damage can affect many parts of the brain and cause problems with memory, thinking, and behavior. While aging is a factor that increases the risk of developing Alzheimer's, scientists suggest that genes and unhealthy habits (e.g., a poor diet, lack of exercise, and smoking) may also increase the risk of developing the disease.

Unfortunately, there is no cure for Alzheimer's disease, only treatments to manage symptoms. Some medications can help slow the progression of the disease and improve the quality of life for people with Alzheimer's. However, as the disease progresses, it becomes more difficult to treat and can ultimately lead to death.

Photobiomodulation is a non-invasive light-based therapy that harnesses invisible wavelengths of light energy to stimulate cellular function and promote healing. Recent studies have shown that it reduces the accumulation of amyloid plaques in the brain in mice. It may be a promising means of improving cognitive function. Photobiomodulation may be able to slow the progression of Alzheimer's disease by irradiating the brain with light of specific wavelengths. Small-scale clinical trials in the United States have already suggested efficacy for Alzheimer's disease patients (https://pubmed.ncbi.nlm.nih.gov/31050950/), and larger confirmatory trials are planned (https://clinicaltrials.gov/ct2/show/NCT04418505).

The device used in this study has been approved for sale by the U.S. Food and Drug Administration as a "general wellness device" due to its high safety and ability to be used at home, but its effectiveness for specific diseases has not yet been verified. In addition, no photobiomodulation device has been approved for the treatment of dementia in any country, including other countries. This study will evaluate whether photobiomodulation improves cognitive decline and promotes brain health in patients with Alzheimer's disease and mild cognitive impairment.

3. About this study

Research title: "Randomized, double-blind, parallel-group comparative study of photobiomodulation for patients with dementia or mild cognitive impairment"

| The clinical study we are requesting your participation in is being conducted in accordance with the provisions of the Clinical Research Act and is considered to be a study using unapproved/off-label ^* drugs.^ (*Off-label refers to the use of drugs or medical devices approved by the Minister of Health, Labour and Welfare as available for use in Japan in a manner other than that approved by the Minister of Health, Labour and Welfare.) |
| --- |

This study was approved by the Clinical Research Review Board of the National Center of Neurology and Psychiatry, and the implementation plan was submitted to the Minister of Health, Labour and Welfare. The implementation of the study was approved by the head of the medical institution conducting the study.

# 4. Method of this study and period of participation

## 1) Study participation criteria

To participate in this study, we ask that you meet all the following criteria:

Main selection criteria

1) People between 60 and 90 years old at the time of obtaining consent

2) Those who have visited our hospital before

3) Those who have been diagnosed with mild Alzheimer's disease or mild cognitive impairment due to Alzheimer's disease at our hospital.

4) Those with an MMSE score between 15 and 30

5) Participants have a study partner (18 years of age or older, blood relationship not required) who lives with them or spends more than 10 hours a week with them and regularly monitors the participants' condition, including the equipment used in this study. It is advisable not to change study partners during participation in the study.

6) Participants or study partners must be able to use a smartphone or personal computer to contact by email and fill out questionnaires.

7) Your dementia medication is stable (no changes for 4 weeks or more)

8) Those who are not planning to undergo any major changes in their environment, such as hospitalization, travel, or moving, within 12 weeks after the start of the study.

9) People who can consent to this clinical study

In addition, if any of the following conditions apply to you, you will not be able to participate in this study.

Main exclusion criteria

1) Those whose doctor has determined that the cause of their cognitive decline is due to causes other than Alzheimer's disease (e.g., mental disorders, intellectual disabilities, etc.)

2) Those who have not had a brain scan (CT or MRI) within the past year, or those who, based on brain image findings, are deemed by a doctor participating in the study to be inappropriate for the use of brain stimulation devices.

3) Those who plan to participate in other clinical trials or clinical studies within the next 12 weeks

4) Other individuals who are deemed inappropriate for participation by a doctor participating in the study.

In addition to the above, your doctor will determine whether or not you can participate based on the results of your examination and tests. In some cases, you may not be able to participate in the study even after you have given your consent. Also, please note that if your doctor determines that it is difficult for you to participate in the study, even if you are in the middle of participating in the study, your participation may be discontinued.

## 2) Drugs, medical devices, and treatments used in the research

The following medical devices will be used in this study:

| name | Vielight Neuro Gamma ( Vielight , Canada ) |
| --- | --- |
| LED specifications | Light source, LED module: 810nm LED (100mW/cm2 ^x^ 3, 75mW/cm2 ^x^ 1)  Light source, nasal applicator: 810 nm LED (25 mW /cm ^2^ )  Frequency: 40 Hz  Duty cycle: 50% |
| Stimulus Specifications | Treatment Time: 20±0.1 minutes with automatic shut-off  Low Power Indicator: <4.2V  Power adapter: AC input: 100-240V, 0.5A , 50 /60Hz  DC output: 5V , 2.0A |
| How to wear | See the figure below ( <https://www.vielight.com/wp-content/uploads/2019/08/Neuro-placement-sheet-2019-A-fin.pdf>) |

The devices used in this study include a sham device that looks identical to the devices mentioned above but does not actually stimulate the participants. The computer will determine which group participants will be in, so participants will not be able to choose. Since it is expected that there will be no direct stimulation with any of the devices used in this study, participants will be asked to predict which group they think they have been assigned to at the end of the test.

## 3) Research period and schedule

In this study, in order to monitor your health condition during the study, you will be asked to regularly report your condition via the Internet.

This product can be used at home once a day at any time of your choice (we recommend using it at the same time each day, such as in the morning or before going to bed, so that you will not forget whether you have used it or not). Each session lasts 20 minutes, after which it will automatically turn off. In principle, this product should be used three times a week, but if you use it for six consecutive days, you must set aside at least one day of non-use. Please avoid using it more than twice a day or for more than seven consecutive days. You will also be asked to report on the usage of this product.

It will take about three months to complete. During that time, we will not only check the effectiveness of the product, but also monitor the participants' medical conditions and safety.

Schedule of this study

| item | Pre-observation period | Intervention Period | | | | Observation period |
| --- | --- | --- | --- | --- | --- | --- |
| Day (D) | 7 days or more before | Start date of use | Every week | Every 4 weeks | After 12 weeks  /When cancelled | 4 weeks after last dose/discontinuation |
| Outpatient/remote care ^*1^ | Outpatient | Outpatient | Remote | Remote | Outpatient | Remote |
| Consent | ○ |  |  |  |  |  |
| Background information *^2^ | ○ |  |  |  |  |  |
| consultation | ○ | ○ |  |  | ○ |  |
| MMSE | ○ *^3^ | ○ |  |  | ○ |  |
| ADAS-Cog |  | ○ |  |  | ○ |  |
| CDR |  | ○ |  |  | ○ |  |
| C GI/PGI |  |  |  | ○ | ○ | ○ |
| D EMQOL |  | ○ |  | ○ | ○ | ○ |
| N PI |  | ○ |  | ○ | ○ | ○ |
| ZBI |  | ○ |  | ○ | ○ | ○ |
| A DL dictionary |  | ○ |  | ○ | ○ | ○ |
| Satisfaction Survey |  | ○ |  | ○ | ○ |  |
| Check usage status |  | ○ | ○ | ○ | ○ |  |
| Combination Therapy Report |  | ○ | ○ | ○ | ○ | ○ |
| Equipment Use |  |  |  |  |  |  |
| Investigation of side effects |  |  |  |  |  |  |

*1: In remote evaluation, the patient does not visit the clinic; instead, questionnaires are filled out and contact is made over the Internet (including by e-mail).

*2: We will investigate your gender, age, illness history, past illnesses and treatments, current illnesses and treatments, allergies, etc.

*3: If MMSE is performed in an outpatient or hospital ward setting within one month of starting administration, the results can be used as is.

## 4) Regarding the tests to be conducted

<Tests to be performed in this study>

In this study, the following tests will be performed:

- MMSE: A simple test that evaluates overall cognitive function. It takes about 5-10 minutes to complete.
- ADAS-Cog: This is a test to evaluate overall cognitive function in dementia such as Alzheimer's disease. The test takes about 40 minutes.
- CDR: This is a test that comprehensively assesses cognitive function and daily living function based on the results of examinations and other tests. The standard test time is 20 minutes, excluding other tests.
- CGI-I/PGI-I: A subjective evaluation of how much improvement researchers and participants feel compared to the start of the study. The evaluation takes about 2 minutes.
- DEMQOL/DEMQOL-Proxy: This is a test that comprehensively asks participants about their quality of life. The test takes about 10 minutes.
- NPI-Q: This is a test that evaluates mental and behavioral symptoms other than cognitive function seen in dementia. The test takes about 20 minutes.
- ZBI: This is a test to evaluate the degree of care burden. The test takes about 10 minutes.
- ADL Assessment Sheet: This is a test to evaluate the ability to perform daily activities. The test takes about 10 minutes.
- Satisfaction assessment questionnaire: This is a questionnaire to evaluate the satisfaction with the equipment used in this study. The test takes about 1 minute.
- Blinding questionnaire: This is a questionnaire to predict whether the device used in this study is the real device or the sham stimulation period. The test takes about 1 minute.

In addition, you will be asked to regularly report on your experience using the devices used in this study, whether there have been any changes to your treatment outside of this study while you are using them, and any adverse events that occur during their use.

# 5. About the burdens, risks, and benefits that may arise from participating in the study

## 1) Possible burdens and risks

### (1) Illness or malfunction

Since the devices used in the study irradiate light energy, it is expected that the light energy will be converted into other energy such as heat in hair, scalp, skull, mucous membranes, etc. that are in the path of the light energy. To date, no adverse events (including serious ones) thought to be caused by the devices used in the study have been reported, but there is a possibility that a slight fever may be felt. In addition, the safety of using the devices used in the study beyond the appropriate conditions of use (20 minutes once a day, 3 to 6 days a week) has not been evaluated, so unexpected risks may arise when used outside of these conditions.

The Clinical Research Act defines "diseases, etc." as illnesses, disabilities, deaths, or infectious diseases suspected to be caused by drugs administered because of participating in clinical research or by malfunctions of medical equipment. In the event of an illness, etc., the principal investigator is obligated to report it to the administrator of the medical institution conducting the research, to notify the principal investigator, and to report it to other principal investigators.

### (2) Other burdens and disadvantages

If you take part in this study, you may be required to visit the clinic more frequently than you would in a general practice, and you may be asked to answer more questions that would not be asked in a general practice setting.

## 2) Expected Benefits

If you participate in this study, the product may slow or improve the progression of symptoms of dementia, including cognitive function, and may also benefit future dementia patients.

## 3) When to discontinue research

1) If you discover after participating in the study that you should not have participated in the study

2) If you decline to participate in this study

3) If you withdraw your consent to participate in this study.

3) When the principal investigator or co-investigator determines that the study needs to be discontinued based on medical judgment.

4) If you are unable to visit the hospital due to moving, being transferred to another hospital, etc.

5) If the principal investigator or other person in charge of the study decides to discontinue the entire study

# 6. Other treatment methods

of February 2024, four types of oral medications (donepezil, rivastigmine, galantamine, and memantine) have been approved in Japan as pharmacological therapy for patients with Alzheimer's dementia, but three types of medications other than memantine are effective for patients with mild Alzheimer's dementia, who are the subjects of this study. In addition, lecanemab, an intravenous amyloid antibody drug ( a drug that acts on and reduces beta amyloid, which causes Alzheimer's disease), has been approved for patients with mild cognitive impairment and mild dementia due to Alzheimer's dementia. As a non-pharmacological therapy, lifestyle improvements such as improving exercise volume and nutrition are widely used because they have no noticeable risks, but neither treatment is thought to completely stop the progression of dementia itself. There are no restrictions on the use of pharmacological or non-pharmacological therapy in this study, but if you start a new treatment during your participation in this study, please report it.

# 7. Financial burden associated with participating in this study

If you participate in this study, we will give you 3,000 yen at the time of the final evaluation to help reduce the burden of your participation.

# 8. Withdrawal of consent after participating in the study

Please decide of your own free will whether to participate in this study. If you decline to participate in this study, you will not suffer any disadvantages. Furthermore, even if you have agreed to participate in the study and the study has begun, you can stop participating at any time, and in that case, you will not suffer any disadvantages. However, once the entire study has been completed and the results have been published, even if you wish to withdraw your consent to participate in the study, it will be difficult to accommodate your request, so we will not be able to accept your request to withdraw.

# 9. Handling of personal information

If you participate in this study, the research data you provide, such as samples and medical information, as well as information that could identify you, such as your name, will be anonymized and managed by assigning a research code so that individuals cannot be identified.

For this study, an anonymization officer will create a correspondence table linking your name with a research number for the purpose of withdrawing your consent to participate in the study, matching medical information, etc., and this will be securely managed by a personal information manager.

In addition, to check whether this clinical study is being conducted appropriately, your information may be accessed by those involved in the clinical study (those authorized to do so by the principal investigator), the Ministry of Health, Labor and Welfare and its related organizations, and the Clinical Research Review Board. However, these parties are required to maintain confidentiality, so your personal information will never be misused.

If the results of the clinical research are made public, the personal information of the subjects of the clinical research will be protected.

# 10. Storage and disposal of samples and information, and secondary use

Samples obtained through this study will be kept as records stipulated in Article 12 of the Clinical Research Act for five years from the date of completion of the study. The final ownership of the data will be that of the principal investigator.

Your data may be used in future research other than this study, but this is not currently planned and will not pose any additional risks to you. If such research is conducted, it will be reviewed again by the Center's ethics committee or clinical research review committee, and after obtaining approval from the Center, your consent to the conduct of another study or your willingness to cooperate with the study will be confirmed through disclosure of information before it is conducted.

# 11. Conflicts of interest regarding research funding sources and researchers

A "conflict of interest" is a situation in which external financial interests, such as falsification of research data, favoritism towards certain companies, or continuation of research when it should be discontinued, may give rise to concerns from third parties that the research is not being conducted fairly and appropriately.

This study is funded by the Japan Society for the Promotion of Science (JSPS) Grants-in-Aid for Scientific Research (Academic Research Grant Fund Grant) Young Researchers (Research topic: Photobiomodulation in patients with dementia and mild cognitive impairment, Research representative: Yuma Yokoi, Subsidy period: FY2023-FY2025), and does not use funds from any specific company. In addition, the principal investigator, sub-investigator, statistical analysis manager, research supervisor, and their spouses and other family members have no financial or employment relationship with Vielight, the manufacturer of Vielight Gamma used in this study . Therefore, the researcher plans and conducts the study independently of companies, etc., and there is no influence on the research results and analysis. Conflicts of interest related to this study will be reviewed by the Clinical Research Review Committee and managed by the principal investigator (in the case of multi-center collaborative research, the principal investigator).

# 12. Provision of medical care after the study is completed

After the study ends, you will receive treatment tailored to your condition. You will not be able to use any of the devices used in this study after the 12-week evaluation.

# 13. Handling of research results from those who participated in the study

The study results of those who participated in this study will be compiled and sent to you electronically.

# 14. Compensation for health damage caused by research participation

Establishing new medical technologies for drugs (and medical devices) through clinical research is essential for the development of medicine and healthcare. However, even if clinical research is conducted correctly with the utmost care, it is said to be difficult to completely prevent the occurrence of health damage caused by drugs (and medical devices). In recent years, emphasis has been placed on responding to health damage caused by drugs and medical devices from the perspective of protecting people who cooperate with research in good faith for the development of medicine and healthcare from the occurrence of health damage.

This study will be scientifically planned and conducted carefully, but if you experience any unusual symptoms or physical discomfort while participating in this study, please inform your doctor immediately. Appropriate measures and treatment will be taken immediately. If any tests or treatment are required, the costs will be paid using your health insurance, just like regular medical care.

Please note that understanding the contents of this information document and agreeing to participate in this clinical study does not mean that you waive your right to claim compensation for health damage.

# 15. Disclosure of research information

　An outline of this research will be registered in a public database ^*^ maintained by the Ministry of Health, Labor and Welfare before the start of the research, and the content will be updated according to changes in the research plan and the progress of the research. When the research is completed, the results of the research will be registered.

The results of this research will be presented only in academic forums, such as conference presentations and papers.

* Clinical research submission and publication system jRCT (Japan Registry of Clinical Trials) https://jrct.niph.go.jp/

# 16. If you wish to obtain or view materials regarding the research plan and research methods

You may view the research plan and materials regarding the research methods, to the extent that it does not interfere with the protection of personal information of other people participating in the research and the securing of the originality of the research. If you wish to do so, please contact "19. Contact information for inquiries regarding this research. "

# 17. About the Clinical Research Review Committee that reviewed this study

The Clinical Research Review Committee (hereinafter referred to as the Committee) is certified by the Minister of Health, Labor and Welfare, and will investigate and deliberate from a scientific and ethical perspective whether there are any issues with patient human rights or safety. The Committee is composed of medical or healthcare professionals, legal experts or bioethics experts who understand the protection of subjects in clinical researchers and the respect for human rights in the medical or healthcare field, and members of the public who have no vested interest in this research or the medical institution. If you have any inquiries regarding the committee's procedures, list of committee members, and content of deliberations related to this research, they are posted at the URL below.

Clinical Research Review Committee

Name: National Research and Development Agency　National Center of Neurology and Psychiatry Clinical Research Review Committee

Established by: National Research and Development Agency　President, National Center of Neurology and Psychiatry

4-1-1 Ogawa Higashicho, Kodaira City, Tokyo

Information on how to view the materials is available at the following URL:

［URL］： https://ninteicrb.ncnp.go.jp/

# 18. The implementation structure of this study, the names of the medical institutions conducting the study, and the name of the principal investigator

Name of main medical institution: National Center of Neurology and Psychiatry, National Research and Development Agency

Name and title of principal investigator: Takuma Inagawa ( Psychiatric Department)

# Contact for inquiries regarding this research

If you or your family have any questions or concerns about this study, please feel free to contact the contact point at the end of this information document. Please note that we may not be able to respond to your inquiry due to reasons such as protecting the personal information of other study participants or the researchers' intellectual property rights.

In addition, if you have experienced any inconvenience in the conduct of this research or wish to file a complaint, please contact the complaints desk.

○Contact point for inquiries regarding this research

Postal code 187-8551

4-1-1 Ogawa Higashicho, Kodaira City, Tokyo

National Center of Neurology and Psychiatry

　　　　　　 Telephone number: 0 42-341-2711

Affiliation /Title　Psychiatric Department

Name: Takuma Inagawa

○Complaint Desk

Postal code 187-8551

4-1-1 Ogawa Higashicho, Kodaira City, Tokyo

National Center of Neurology and Psychiatry

Clinical Research Review Committee Secretariat

　　　　　　 Email: crb-jimu@ncnp.go.jp

Consent form for participating in the study

Research Principal Investigator: Takuma Inagawa

and understand the following matters regarding the "Research Project Title: Randomized, double-blind, parallel-group comparative study of photobiomodulation for patients with dementia or mild cognitive impairment" using the explanatory document. I will participate in this study of my own free will.

Items that were explained and understood

□ 1. Purpose and significance of this study (Explanatory document, item 2)

□2. Method of carrying out this study and period of participation (explanation document) Item 4)

□ 3. Information about the burdens, risks, and benefits that may arise from participating in the study (explanation document) Item 5)

□4　Other treatment methods (Information sheet item 6)

□5. Financial burden associated with participating in the study (explanation document) Item 7)

□ 6　Voluntary participation in the study and withdrawal of consent after participation (Information document, item 8)

□6-1 Even if you agree to participate in this study, you can withdraw at any time

□6-2 I will not suffer any disadvantages in terms of treatment if I choose not to participate in this study or if I withdraw my consent.

□ 7. Handling of personal information, etc. (Explanatory document Item 9)

□8. Storage and disposal methods for samples and information, and secondary use (Explanatory Document, Item 10)

□9. Regarding the source of research funding and conflicts of interest of researchers, etc. (Explanatory document) Item 11)

□10 Regarding provision of medical care after the study is conducted (Explanatory document Items 1 and 2 )

□ 11 Regarding the handling of research results of those who participated in the study (explanation document Items 1-3)

Provide information when important health findings are discovered by chance or when important knowledge is obtained.

□ Yes □ No

□1 2 Compensation for health damage caused by participating in the study (Explanatory document Items 1-4)

□13 Regarding disclosure of information regarding research (Explanatory Document, Item 15)

□14 If you wish to obtain or view materials regarding the research plan and research methodology (explanation document) Item 16 )

Signature field

Date of consent: 20th year, month, day

Signature (autograph)

Study Partner Signature

Date of consent: year, month, day

Signature (signature) (relationship)

Signature of principal investigator or co-investigator

When obtaining the subjects' consent for this study, I explained the study to them based on a written explanation.

Date of explanation: year, month, day

Signature (autograph)
